# Microbial Isolates from Vegetable Foreign Bodies Inhaled by Dogs

**DOI:** 10.1155/2018/3089282

**Published:** 2018-11-27

**Authors:** Sara Flisi, Manuel Dall'Aglio, Costanza Spadini, Clotilde Silvia Cabassi, Fausto Quintavalla

**Affiliations:** ^1^Department of Veterinary Science, University of Parma, Infectious Diseases Unit, Parma 43126, Italy; ^2^Veterinary Practitioner, Parma 43100, Italy; ^3^Department of Veterinary Science, University of Parma, Clinical Medicine Unit, Parma 43126, Italy

## Abstract

Grass-seed inhalation is a common problem in canine patients, in particular during summer months, migrating in upper and lower respiratory tract. Grass awns can harbor bacteria and fungi, causing grass seeds foreign body-related disease (GSFBD). Aim of this study was to investigate the aerobic microbial flora isolated from grass awns extracted from 41 dogs with GSFBD and the antibiotic susceptibility of the isolated bacterial strains. Fifty-four grass awns were localized with diagnostic imaging tests and removed by endoscopy from respiratory tract. The most frequent localizations were in the left nostril and the right hemithorax. Only one grass awn was extracted from each patient except in 7 that had more than one. Bacteriological and mycological cultures, strains identification, and antibiotic susceptibility tests were performed. One or more bacterial strains were isolated from all grass awns. Fungal strains were isolated only in 4 cases. S*taphylococcus *sp. was the most frequent isolate in the upper respiratory tract (36.8%), while* E. coli* (24.4%) was the most frequent isolate in the lower tract. Fluoroquinolones and Doxycycline were the most effective antibiotics, while resistance was observed against Gentamicin (>93%), Cefapirin, and Clindamycin (>80%). These data are relevant in relation to the use of these antibiotics in both animals and humans, for the risk of transmission of antibiotic resistant bacteria or resistance genes.

## 1. Introduction

Cases of grass awns accidentally inhaled by the dog after a walk in the countryside or in the city green areas are very frequent during summer months. These are seeds of several species of Graminaceae, most frequently* Hordeum murinum *commonly named “wild barley” and* Avena fatua* or “wild oats” [1] that can be considered the major cause of grass seeds foreign body-related disease (GSFBD). However, different species of grass awns are reported regionally:* Hordeum jubatum, H. glaucum, H. leparium* or “foxtail” grass, spear grass (*Austrostipa* spp.), brome grass (*Bromus* spp.), and* Setaria* spp. Usually the front part of the spike penetrates through the skin, in particular dorsal interdigital webs, or body orifices as the external ear canal, oral cavity, nostril, conjunctiva, and foreskin. The shape of the grass awn and the presence of anterior barbet florets cause forward migration of this foreign material [2, 3]. Wood and plant materials can be directly irritating and evoke a sterile or septic foreign body reaction, as they host bacteria and/or fungi [2]. Once inhaled, grass awns may stop in the nose and nasal sinuses; in other cases, they can migrate through oral cavity and then in trachea to bronchioles. This could cause tracheobronchitis, focal pneumonia, or penetration of the lung resulting in pleural space disease (chronic pleuritis, pneumothorax, and pyothorax). In a second time, once deflected by the diaphragm, grass seeds can migrate in different locations, even far from the first: peritoneal cavity [4], sublumbar muscles [5], lumbar vertebrae [2, 6–8], and so on. Clinical signs of inhalation of grass seed can be acute or chronic: sneezing, cough, dyspnea, and pyrexia of variable duration. Often, abscesses or granulomatous tissues are detectable at the site of migration. An inflammatory leukogram with a left shift and toxic cells should further raise the index of suspicion [9].

The importance of GSFBD has been documented in northern America, Europe, and Australia [10]. The prognosis for affected dogs is good if the foreign bodies can be accurately localized with diagnostic imaging tests (radiography, ultrasonography, sonography, computed tomography, and magnetic resonance imaging) and completely removed [1, 9, 11]. Among these, endoscopy still is the most effective, allowing diagnosis and direct removal [12, 13].

The first aim of the present work was to identify in a canine population the most frequent respiratory localizations of inhaled foreign vegetal bodies. The other aim was to investigate the microbial aerobic flora isolated from grass awns extracted from dogs with GSFBD and the antibiotic susceptibility of the isolated bacterial strains, in order to evaluate the relative most effective antibiotics and the presence of resistant bacteria.

## 2. Materials and Methods

This work is a retrospective study that takes into consideration 41 dogs of different breed, age, and sex (Tables 1 and 2), presented to the Veterinary Teaching Hospital of the Department of Veterinary Science (University of Parma, Italy) during the period June 2016-September 2017. All had a history of respiratory clinical signs for at least two weeks. At presentation, moderate to severe respiratory distress is usually present with coughing and/or sneezing, nasal discharge, and reduced performance. Despite administration of a first empirical treatment with antibiotic and anti-inflammatory (corticosteroids or NSAIDs) by Veterinary Practitioners, clinical signs persisted. Ultrasonographic and radiographic insights allowed making a diagnosis of GSFBD. After localization of the vegetable foreign body by diagnostic imaging tests (radiography, ultrasonography), each animal was anesthetized for endoscopic extraction of the foreign body. A high standard of veterinary care was adopted: patients were managed with the best ethical standards concerning animal welfare and only after signature of informed client consent, following Directive 2010/63/EU.

### 2.1. Endoscopic Procedure

Before the endoscopic examination, each patient was sedated with an intramuscular combination of Dexmedetomidine (5 *μ*g/kg) and Butorphanol (0.2 mg/kg); then intravenous administration of Propofol (4 mg/kg) has been used for the anesthesia induction.

General anesthesia was maintained with isoflurane 2% vaporized in oxygen. Foreign bodies extraction from nasal cavity was performed through anterograde rhinoscopy using a LED light source (Storz Nova led 150, Storz Medical AG, Tägerwilen, Switzerland), a camera (Storz Telecam Pal 202100-20, Storz Medical AG, Tägerwilen, Switzerland), an optics (Storz Hopkins II 2.7mm 30°, Storz Medical AG, Tägerwilen, Switzerland) and Hartman clamp. Foreign bodies in the lower respiratory tract were removed with tracheobronchoscopy, using a pediatric gastroscope (Fujinon EG-270N5 pediatric gastroscope, Fujinon, Japan) connected to a processor (Fujinon video-processor EXP-2500, Fujinon, Japan). To avoid contaminations and to not alter laboratory analyses, clumps were sterilized with autoclave at 121°C in double envelop, while other instrumentation was sterilized through immersion in 2% peracetic acid solution for 10 minutes and then washed with sterile saline. Grass awns were maintained at 4°C in a sterile tube and sent to the laboratory within 1 hour after extraction.

### 2.2. Cultural Examination and Antimicrobial Susceptibility Test

After retrieval, vegetable foreign material was immediately plated onto Tryptose agar (Difco, Sparks, USA) containing 5% bovine erythrocytes, MacConkey agar (Difco, Sparks, USA) and Sabouraud agar (Difco, Sparks, USA) with a sterile single-use tweezer to carry out aerobic bacterial and fungal culture and antibiotic sensitivity testing. Agar plates were incubated at 37°C in aerobic conditions for 18-24 hours for bacteria and 48 hours for yeasts.

After incubation, bacterial growth was evaluated and colonies were isolated and amplified when necessary. Identification of bacterial strains was based on growth and colony characteristics, Gram staining, cellular morphology, catalase and oxidase reactions, and API biochemical test system (bioMérieux, France), as well as conventional biochemical tests [14]. Catalase positive Gram positive strains (*Staphylococcus* sp.) were distinguished on the basis of the coagulase reaction in* Staphylococcus *coagulase positive and* Staphylococcus* coagulase negative (CoNS) followed by species identification by API Staph biochemical test.

Catalase negative Gram positive strains were identified by API Strep biochemical test, while Gram negative aerobic strains species identification was carried out by API 20E and API 20NE for oxidase negative and oxidase positive strains, respectively.

Yeasts growth on Sabouraud agar was also evaluated. Identification of fungal strains was based on growth and colony characteristics and cellular staining (methylene blue) and morphology, compared to the literature [14].

For each bacterial strain, antimicrobial susceptibility test was performed by agar disk diffusion methods [15], according to CLSI guidelines [16]. The list of tested antibiotics and their concentrations is reported below: Amikacin (30 *μ*g), Amoxicillin + Clavulanic acid (30 *μ*g), Cefadroxil (30 *μ*g), Cefazolin (30 *μ*g), Cefotaxime (30 *μ*g), Cefovecin (30 *μ*g), Ceftazidime (30 *μ*g), Ceftriaxone (30 *μ*g), Clindamycin (2 *μ*g), Doxycycline (30 *μ*g), Florfenicol (30 *μ*g), Gentamicin (10 *μ*g), Oxytetracycline (30 *μ*g), Trimethoprim + Sulfamethoxazole (25 *μ*g) (all produced by Oxoid, Basingstoke, Hampshire England); Ampicillin (25 *μ*g), Cefapirin (30 *μ*g), Metronidazole (5 *μ*g) (all produced by Mast Diagnostics, Merseyside, UK); Enrofloxacin (5 *μ*g), Marbofloxacin (5 *μ*g) (both produced by Biolab, Budapest, Hungary).

Antibiotic medium 1 agar plates (Difco, Sparks, USA) were incubated at 37°C in aerobic conditions for 24 hours and then the diameters of growth inhibition zones were measured and compared with those reported by CLSI guidelines [16] to determine bacterial susceptibility or resistance.

### 2.3. Statistical Analysis

Statistical analysis was performed through the chi-square test obtained through free online software [17]. Statistical correlation was evaluated between grass awn localization and different patient's parameters (age, sex, and breed).

## 3. Results 

Tables 1 and 2 show localization of grass awns extracted by endoscopy, from upper and lower respiratory tract, respectively, in addition to age, sex, and breed of each patient.

Patients were aged 4 months to 14 years (average 3.96 years). 43.9% of examined dogs were young, aged 0-2 years (18/41); 43.9% of examined dogs were young adult, aged 3-7 years (18/41); and 12.2% of examined dogs were mature adult, aged > 7 years (5/41). Statistical evaluation among the three age groups showed no significant differences (p = 0.94).

For the localization of the grass awns, we evaluated data concerning two sites of extraction: upper and lower respiratory tract. In the present study, on a total number of 41 dogs and 54 grass awns, 25 were extracted from nostrils (46%) and 29 from bronchi and lungs (54%).

**Figure 1:**
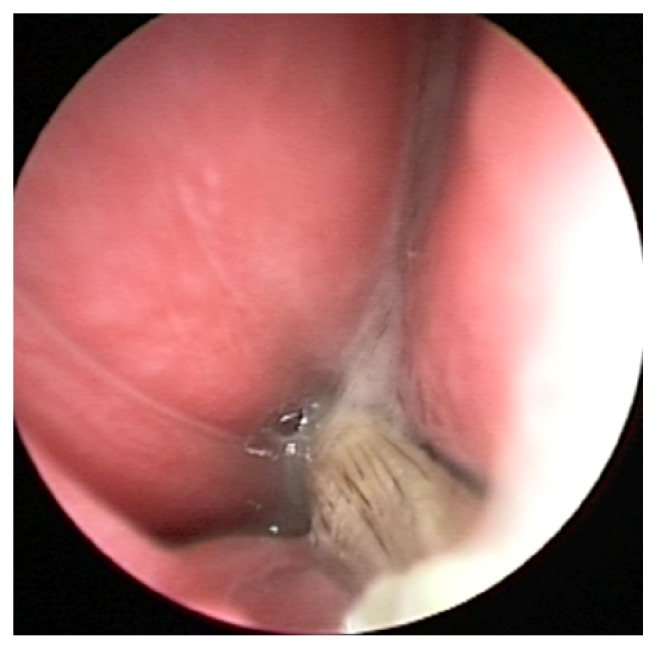
Grass awn in dog nostril during endoscopy extraction.

In detail, from upper respiratory tract, grass awns were extracted from 24/41 patients for a total number of 25 awns (Figure 1 and Table 1). Grass awns were found more frequently in male patients (17/24) (70.83%). A single grass awn was retrieved from all patients but one. Two grass awns were found in patient number 7, one within each nostril. The most frequent localization was in the left nostril (14/25, 56%). Grass awns were extracted from dorsal meatus in 5 cases (two from the right and three from the left nostril), 13 from middle meatus (8 from the right and 5 from the left nostril), and 7 from ventral meatus (one from the right and 6 from the left nostril).

**Figure 2:**
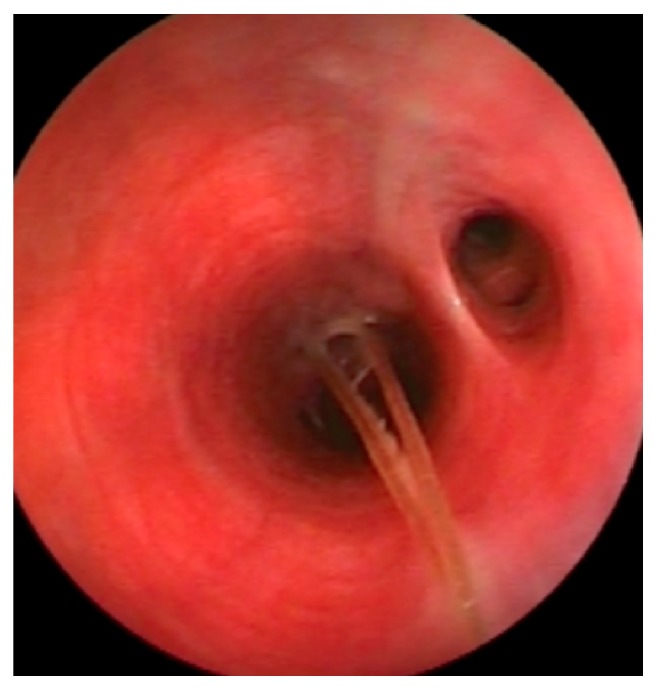
Grass awn in a bronchus during endoscopy extraction.

From lower respiratory tract grass awns were extracted from 17/41 patients for a total number of 29 awns (Figure 2 and Table 2). Also in this case male dogs were most involved (11/17, 64.7%). However, in our overall population sample grass awns localization after inhalation is not related to animal sex (p = 0.68).

In most cases, only one grass awn was extracted, but 6 animals had more than one. In particular:

- Cases numbers 2, 3, and 9 had two awns in the same localization;
- Case number 5 had three awns, one in left and 2 in right hemithorax;
- Case number 15 had four awns in the right hemithorax, three in the middle lobar bronchus and one in the caudal lobar bronchus;
- Case number 17 had five awns, three in the right and two in the left hemithorax.

The most frequent localization was the right hemithorax (22/29, 76%), in particular the right caudal lobar bronchus. Grass awns were extracted from caudal lobar bronchus in 14/29 cases, 10 from the right hemithorax and 4 from the left. Two awns were from the left cranial bronchus, 2 from the right accessory bronchus, and 7 from the middle one (6 from the right and one from the left hemithorax). Four grass awns were extracted from lung, always from the right caudal lobe.

In the present study, 39% of the patients were hunting or working dogs (16/41). Seven of these hunting dogs had grass awns in the upper respiratory tract (*vs *17 of the other dog breeds) and nine in the lower (*vs* 8 of the other), but this difference was not statistically significant (p = 0.12).

### 3.1. Bacterial Isolates and Antibiotic Susceptibility

In general, bacterial growth was heavy in all cases. One or more aerobic bacterial strains were isolated from all extracted grass awns. Polymicrobial infections were reported in 13 of 24 cases of GSFBD from the upper respiratory tract and in 14 of a total number of 17 cases of GSFBD from the lower respiratory tract.

Bacterial isolates can be classified into primary pathogens and opportunistic or secondary bacterial strains. More specifically,* Staphylococcus *sp. (26,6%),* E. coli *(22,8%)*, Pseudomonas *sp. (13,9%),* Pasteurellaceae *(5%),* Streptococcus *sp. (1,3%), and* Burkholderia* sp. (1,3%) can be considered as primary pathogens, while others may be considered opportunistic.

In the upper respiratory tract, 71% aerobic bacterial isolates were primary pathogens, while in the lower respiratory tract they were 61%. The most frequent microorganism in the upper respiratory tract was* Staphylococcus* sp. (36.8%), while* E. coli *was the most frequently (24.4%) isolated from the lower respiratory tract.

Antimicrobial susceptibility was evaluated for each bacterial isolate. Detailed results are reported in Tables 3 and 4. Based on the antimicrobial susceptibility tests, the most effective antibiotics were as follows:

- Marbofloxacin: overall 88,6% bacteria were susceptible, in particular 88.5% primary pathogens and 85,2% secondary ones
- Enrofloxacin: overall 79.7% bacteria were susceptible, in particular 76,9% primary pathogens and 85,2% secondary ones
- Doxycycline: overall 60.8% bacteria were susceptible, in particular 57.7% primary pathogens and 66,7% secondary ones

High resistance percentages were reported against Gentamicin (93.7% of isolates, among which 92.7% were primary and 95.8% were secondary pathogens). More than 80% of the isolated bacterial strains were resistant to Cefapirin or Clindamycin, both primary and secondary pathogens.

### 3.2. Fungal Isolates

Fungal strains were isolated only from four samples, 3 in the upper respiratory tract and 1 in the lower. In particular,* Aspergillus* sp. was isolated in case number 2 by upper respiratory tract, and* Candida *sp. was isolated in cases numbers 19 and 20 by upper tract and case number 15 by lower respiratory tract (Tables 3 and 4).

## 4. Discussion

To date, in literature only few reports regarding GSFBD can be found, often relating to a limited number of clinical cases. Moreover, data regarding bacteriological investigations on the vegetable foreign bodies are further limited. In our work, we evaluated a population sample of critical importance (41 dogs) and performed an extended evaluation on the aerobic bacterial isolates and their antibiotic sensitivity.

On the basis of data reported in research articles examining a wider population sample [5, 11], respiratory localization of vegetable foreign bodies is the most frequent and the incidence reach up to 62% in dogs with GSFBD. The nasal localization of grass awns observed in our study appears to be higher than that reported by other authors, considering it a sporadic occurrence in dogs [5]. Conversely, our data regarding localization of most vegetable foreign bodies inside the right lung are in accordance with literature [12, 18–20]. Cerquetella et al. [12] reported that this localization is probably due to the straight angle usually seen between the right principal bronchus and left principal bronchus.

Some authors reported that hunting and working breeds had the highest prevalence because of increased grass awn contact [9, 21]. Mainly grass awn joins dog's respiratory tract during open-mouth breathing associated with exercise [22] or through nostrils for dogs that sniff the soil. Another way of entry into the respiratory tract could be grass awn's migration into the fur moving forward in conjunction with any movement of the animal. The grass awn's wedge follows airflow and her barbs prevent retrograde migration. Nevertheless, our data reported absence of statistical relevance (p= 0.12) for correlation with breed.

In literature, no sex predilection was noted [21]. In our study 68.3% of cases were male, but statistical evaluation (p = 0.68) confirmed the absence of statistical relevance.

Previous studies [1, 12, 21] indicate that young age is a critical hazard feature. In our study, no significant differences were noted (p = 0.94) related to age, though the majority of the examined animals were young.

Plant materials are not inert, as they may harbor bacteria and/or fungi, and may evoke a septic reaction, resulting in chronic infection [2]. There are different studies about nasal normal flora of healthy dogs [23–25], but only a few about bacterial flora conveyed by vegetable foreign bodies into respiratory system. A healthy animal can usually clear bacteria from the airways unless total numbers, high virulence, or concurrent direct injury overwhelms the pulmonary defenses [26]. Lindsey and Pierce reported that lungs harbored aerobic bacteria with a mean concentration of 1.3 x 10^3^ organisms per gram of tissue and 74% of identical bacterial isolates were found in the pharynx of the same animal [27]. In our study the isolated bacteria were common commensal flora of dog's oral cavity; therefore it could be hypothesized that grass awns, extracted from the lungs, passed through this way instead of the nostrils. Indeed, the most isolated bacterial strains from grass awns were* Staphylococcus *sp. and* E. coli,* from upper and lower respiratory tract, confirming Brennan and Ihrke's study [21]. Other bacterial strains frequently isolated in our study were* Pseudomonas* sp.,* Bacillus* sp., and* Pasteurellaceae*, according to these authors [21].

Fungal contamination was observed in some awns extracted by dog's nostrils. They can cause opportunistic infections in case of breaks in the normal mucosal barrier, immunosuppression, and treatment with broad-spectrum antibiotics.* Aspergillus *sp. is reported in literature as the most commonly isolated hyphal fungus from nasal infections in dogs [28]. In our study,* Aspergillus *sp. was isolated only in one case; this sporadic relief is in accordance with data reported by Meler et al. [29].* Candida* species are typically considered commensal organisms and a component of the nasal microbiota of dogs and horses. Nevertheless, our study showed a very low prevalence of* Candida* isolates.

Treatment of GSFBD was performed through removal of the grass awns. The endoscopic technique is the gold standard in the extraction of foreign bodies, for those at both nasal and bronchial level [30]. After the localization and extraction of the foreign bodies, treatment usually entails antibiotic therapy [9]. Lappin et al. [31] suggest that Fluoroquinolones and Doxycycline are the first-line drug options for respiratory tract disease in dogs. In the present study, the vast majority of aerobic bacterial strains were susceptible to Fluoroquinolones and Doxycycline.

Conversely, high resistance percentages against Gentamicin and Clindamycin were reported in our sample study. Our results can be considered relevant in relation to the use of these antibiotics in both animals and humans and consequently to the risk of transmission of antibiotic resistant bacteria or genes [32, 33].

As far as we know, our study is the first report of an extended number of cases focused on the evaluation of aerobic microbial flora isolated from vegetable foreign bodies in the canine respiratory system and its antibiotic sensitivity. The obtained data are robust and we believe that they can represent useful information for the clinical and microbiological management of GSFBD in dogs. Further investigations could take into consideration an implementation of microbiological evaluation, focusing even on anaerobic and fastidious organisms, as suggested by the literature [10].

## Figures and Tables

**Table 1:** Breed, age, and sex of dogs included in the present study and localization of the grass awns in the upper respiratory tract (^*∗*^ F = female; M = male; D = dorsal meatus; Md = middle meatus; V = ventral meatus).

**Case**	**Breed**	**Age**	**Sex** ^**∗**^	**Localization**	
				**Right nostril**	**Left nostril**
**1**	Boxer	2 yrs	M	Md	-
**2**	Mongrel	4 months	F	Md	-
**3**	Chihuahua	2 yrs	F	Md	-
**4**	Mongrel	5 yrs	M	-	D
**5**	Akita Inu	1 yr	M	-	Md
**6**	Jack Russel	4 yrs	M	Md	-
**7**	Dachshund	1 yr	M	V	Md
**8**	Mongrel	13 yrs	F	-	V
**9**	Mongrel	2 yrs	F	Md	-
**10**	Setter gordon	4 yrs	M	-	V
**11**	Cocker	1 yr	M	-	Md
**12**	Shar Pei	8 yrs	M	-	V
**13**	Mongrel	5 yrs	F	-	V
**14**	Half breed	6 yrs	M	-	D
**15**	Shi-Tzu	3 yrs	F	-	V
**16**	Italian Spinone	14 yrs	M	-	Md
**17**	Lagotto romagnolo	1 yr	M	D	-
**18**	Bloodhound	2 yrs	M	D	-
**19**	Jack Russel	2 yrs	M	Md	-
**20**	Mongrel	7 yrs	M	Md	-
**21**	Mongrel	3 yrs	M	Md	-
**22**	Jack Russel	1 yr	M	-	V
**23**	Dachshund	4 yrs	F	-	D
**24**	Pincher	4 yrs	M	-	Md

**Table 2:** Breed, age, and sex of dogs included in the present study and localization of the grass awns in the lower respiratory tract (^**∗**^ F = female; M = male; B = lobar bronchus; C = caudal; Cr = cranial; Md = middle; A = accessory; L = lung; CL = caudal lobe).

**Case**	**Breed**	**Age**	**Sex** ^**∗**^	**Localization** ^**∗**^	
				**Right hemithorax**	**Left hemithorax**
**1**	Deutsch Kurzhaar	10 yrs	M	CB	-
**2**	Weimaraner	1 yr	F	2 CL	-
**3**	Mongrel	3 yrs	M	-	2 CB
**4**	Lagotto Romagnolo	1 yr	M	CL	-
**5**	Jack Russel	4 yrs	M	2 CB	CB
**6**	Bloodhound	4 yrs	M	-	CrB
**7**	Mongrel	6 yrs	M	-	CrB
**8**	Boxer	3 yrs	M	CL	-
**9**	Drahthaar	2 yrs	F	2 CB	-
**10**	English Setter	2 yrs	M	AB	-
**11**	English Setter	3 yrs	M	CB	-
**12**	Bloodhound	8 yrs	F	CB	-
**13**	Mongrel	10 months	M	MdB	-
**14**	Wippet	2 months	F	CB	-
**15**	Doberman	3 yrs	M	CB + 3 MdB	-
**16**	American Bulldog	2 yrs	F	MdB	-
**17**	Mongrel	4 yrs	F	MdB + AB + CB	MdB + CB

**Table 3:** Microbial isolates from vegetable foreign bodies extracted from upper respiratory tract and their antibiotic susceptibility.

**Case**	**Cultured microorganisms**	**Bacterial antibiotic susceptibility**
**1**	*E. coli*	AP, DOX, FFC, OT
**1**	*Pantoea* sp.	AP, DOX, FFC, OT
**2**	*Aspergillus *sp.	
**2**	*Enterobacter cloacae*	ENF, MAR
**3**	*Pseudomonas fluorescens*	MAR
**4**	*E. coli*	CPR, CTX, CAZ, DOX, ENF, MAR, OT
**4**	*Pseudomonas aeruginosa*	CPR, CTX, CAZ, DOX, ENF, MAR, OT
**5**	*E. coli*	AMC, CDX, KZ, CTX, CRO, DOX, ENF, MAR
	*Staphylococcus* coagulase negative (CoNS).	AMC, CDX, KZ, CTX, CRO, DOX, ENF, MAR
**6**	*E. coli*	AK, AMC, AP, CPR, CTX, CVN, CAZ, FFC, MAR
**7**	*Aeromonas hydrophila/caviae*	CTX, CAZ, CRO, DOX, ENF, MAR, OT
**7**	*Staphylococcus aureus *	CTX, CAZ, CRO, DOX, ENF, MAR, OT
**8**	*Proteus* sp.	FFC
**8**	*Staphylococcus pseudintermedius*	FFC
**9**	*Methylobacterium mesophilicum*	AK, AMC, AP, CDX, KZ, CTX, CVN, CAZ, CRO, CD, DOX, ENF, FFC, CN, MAR, OT, SXT
**9**	*Pasteurella pneumotropica*	AK, AMC, AP, CDX, KZ, CTX, CVN, CAZ, CRO, CD, DOX, ENF, FFC, CN, MAR, OT, SXT
**10**	*Staphylococcus* coagulase negative (CoNS)	AK, CRO, CAZ, ENF, OT
**11**	*Bacillus* sp.	DOX, ENF, FFC, MAR
**11**	*Staphylococcus* coagulase negative (CoNS)	DOX, ENF, FFC, MAR
**12**	*Mannheimia haemolytica*	AMC, AP, CDX, KZ, CTX, CVN, CAZ, CRO, DOX, ENF, FFC, OT, SXT
**12**	*Staphylococcus* coagulase negative (CoNS)	AMC, AP, CDX, KZ, CTX, CVN, CAZ, CRO, DOX, ENF, FFC, OT, SXT
**13**	*Staphylococcus aureus*	AMC, CDX, KZ, CTX, CVN, DOX, ENF, FFC, MAR
**14**	*Staphylococcus* coagulase negative (CoNS)	AMC, CDX, KZ, CTX, CVN, DOX, FFC
**15**	*Aeromonas hydrophila/caviae*	AK, CAZ, ENF, MAR, OT
**16**	*Staphylococcus pseudintermedius*	AK, CTX, CAZ, CRO, DOX, ENF, MAR, OT
**16**	*Pseudomonas fluorescens*	AK, CTX, CAZ, CRO, DOX, ENF, MAR, OT
**17**	*E. coli*	AMC, KZ, CTX, CVN, CAZ, CRO, DOX, ENF, FFC, MAR, OT
**18**	*E. coli*	AMC, KZ, CTX, CVN, CAZ, CRO, ENF, FFC, MAR
**18**	*Staphylococcus aureus*	AMC, KZ, CTX, CVN, CAZ, CRO, ENF, FFC, MAR
**18**	*Streptococcus* sp.	AMC, KZ, CTX, CVN, CAZ, CRO, ENF, FFC, MAR
**19**	*Candida* sp.	
**19**	*Staphylococcus pseudintermedius*	AMC, AP, CDX, CPR, KZ, CTX, CVN, CAZ, CRO, CD, DOX, ENF, FFC, MAR, MZ, OT, SXT
**20**	*Candida* sp.	
**20**	*Staphylococcus pseudintermedius*	AMC, AP, CDX, CPR, KZ, CTX, CVN, CAZ, CRO, CD, DOX, ENF, FFC, MAR, MZ, OT, SXT
**21**	*Staphylococcus pseudintermedius*	AMC, AP, CDX, CPR, KZ, CTX, CVN, CAZ, CRO, CD, DOX, ENF, FFC, MAR, OT, SXT
**22**	*Bacillus* sp.	ENF, MAR
**22**	*E. coli*	ENF, MAR
**22**	*Staphylococcus* coagulase negative (CoNS)	ENF, MAR
**23**	*E. coli*	AMC, CTX, FFC, MAR
**23**	*Kluyvera* sp.	AMC, CTX, FFC, MAR
**24**	*Pseudomonas aeruginosa*	AK, CAZ, ENF, MAR

Tested antibiotics: Amikacin (AK); Amoxicillin + Clavulanic acid (AMC); Ampicillin (AP); Cefadroxil (CDX); Cefapirin (CPR); Cefazolin (KZ); Cefotaxime (CTX); Cefovecin (CVN); Ceftazidime (CAZ); Ceftriaxone (CRO); Clindamycin (CD); Doxycycline (DOX); Enrofloxacin (ENF); Florfenicol (FFC); Gentamicin (CN); Marbofloxacin (MAR); Metronidazole (MZ); Oxytetracycline (OT); Trimethoprim + Sulfamethoxazole (SXT).

**Table 4:** Microbial isolates from vegetable foreign bodies extracted from lower respiratory tract and their antibiotic susceptibility.

**Case**	**Cultured microorganisms**	**Bacterial antibiotic susceptibility**
**1**	*Bacillus* sp.	CDX, DOX, ENF, MAR, OT
**1**	*Cryseobacterium indologenes, *	CDX, DOX, ENF, MAR, OT
**2**	*Pasteurella multocida*	AK, AMC, AP, CDX, KZ, CTX, CVN, CAZ, CD, DOX, ENF, CN, MAR, OT, SXT
**3**	*E. coli*	DOX, ENF, FFC, MAR, OT
**3**	*Pseudomonas aeruginosa*	DOX, ENF, FFC, MAR, OT
**4**	*Pasteurella multocida*	AP, CDX, CPR, KZ, CTX, CVN, CAZ, CRO, CD, DOX, ENF, FFC, MAR, OT, SXT
**5**	*E. coli*	AP, KZ, CRO, CD, DOX, FFC, CN, OT, SXT
**5**	*Bacillus* sp.	DOX, ENF, FFC, MAR, OT
**5**	*Proteus* sp.	AK, CDX, CVN, CAZ, CRO, DOX, ENF, FFC, MAR
**6**	*Pseudomonas aeruginosa*	CAZ, MAR, OT
**7**	*Burkholderia cepacia*	CAZ, ENF, MAR
**7**	*Pseudomonas aeruginosa*	CAZ, ENF, MAR
**7**	*Staphylococcus* coagulase negative (CoNS)	CAZ, ENF, MAR
**8**	*Ochrobactrum anthropi*	AMC, AP, CDX, CPR, KZ, CTX, CVN, CRO, CD, DOX, ENF, FFC, MAR, SXT
**8**	*Staphylococcus aureus*	AMC, AP, CDX, CPR, KZ, CTX, CVN, CRO, CD, DOX, ENF, FFC, MAR, SXT
**9**	*Bacillus* sp.	ENF, MAR
**9**	*E. coli*	ENF, MAR
**9**	*Stenotrophomonas maltophilia*	ENF, MAR
**10**	*Bacillus* sp.	DOX, ENF, MAR
**10**	*E. coli*	DOX, ENF, MAR
**10**	*Pseudomonas fluorescens*	DOX, ENF, MAR
**11**	*Bacillus* sp.	DOX, ENF, MAR, OT
**11**	*E. coli*	DOX, ENF, MAR, OT
**11**	*Staphylococcus pseudintermedius*	DOX, ENF, MAR, OT
**12**	*Pseudomonas fluorescens *	CAZ, MAR
**12**	*Staphylococcus* coagulase negative (CoNS)	CAZ, MAR
**13**	*Bacillus* sp.	DOX, ENF, MAR
**13**	*E. coli*	DOX, ENF, MAR
**13**	*Pseudomonas aeruginosa*	DOX, ENF, MAR
**14**	*Bacillus* sp.	AMC, ENF, MAR
**14**	*E. coli*.	AMC, ENF, MAR
**14**	*Staphylococcus* coagulase negative (CoNS)	AMC, ENF, MAR
**15**	*Bacillus* sp.	AK, CRO, DOX, ENF, FFC, MAR, OT
**15**	*Candida *sp.	
**15**	*E. coli*	AK, CRO, DOX, ENF, FFC, MAR, OT
**15**	*Stenotrophomonas maltophilia*	AK, CRO, DOX, ENF, FFC, MAR, OT
**16**	*E. coli*	DOX, MAR
**16**	*Pseudomonas aeruginosa*	DOX, MAR
**16**	*Staphylococcus* coagulase negative (CoNS)	DOX, MAR
**17**	*Bacillus* sp.	ENF, MAR
**17**	*E. coli*	ENF, MAR
**17**	*Staphylococcus* coagulase negative (CoNS)	ENF, MAR

Tested antibiotics: Amikacin (AK); Amoxicillin + Clavulanic acid (AMC); Ampicillin (AP); Cefadroxil (CDX); Cefapirin (CPR); Cefazolin (KZ); Cefotaxime (CTX); Cefovecin (CVN); Ceftazidime (CAZ); Ceftriaxone (CRO); Clindamycin (CD); Doxycycline (DOX); Enrofloxacin (ENF); Florfenicol (FFC); Gentamicin (CN); Marbofloxacin (MAR); Metronidazole (MZ); Oxytetracycline (OT); Trimethoprim + Sulfamethoxazole (SXT).

## Data Availability

The data used to support the findings of this study are included within the article.

## Abbreviations

GSFBD: Grass seeds foreign body-related disease
CLSI: Clinical and Laboratory Standards Institute.
